# Gabapentinoid use and the risk of fractures in patients with inflammatory arthritis: nested case–control study in the Clinical Practice Research Datalink Aurum

**DOI:** 10.1186/s12916-024-03774-5

**Published:** 2024-12-12

**Authors:** Ian C. Scott, Noor Daud, James Bailey, Helen Twohig, Samantha L. Hider, Christian D. Mallen, Kelvin P. Jordan, Sara Muller

**Affiliations:** 1 Primary Care Centre Versus Arthritis, School of Medicine, Keele University, Keele, UK; 2 Haywood Academic Rheumatology Centre, Haywood Hospital, Midlands Partnership University NHS Foundation Trust, High Lane, Burslem, Staffordshire UK

**Keywords:** Rheumatoid arthritis, Psoriatic arthritis, Axial spondyloarthritis, Gabapentinoids, Fracture

## Abstract

**Background:**

Gabapentinoids are increasingly prescribed in inflammatory arthritis (IA), despite no trial evidence for efficacy at managing pain in this population. Observational studies in non-IA populations suggest gabapentinoids are associated with fractures but are limited by methodological heterogeneity/potential residual confounding. Patients with IA generally have an increased risk of fracture so may be particularly vulnerable. We examined the relationship between fractures and gabapentinoids in patients with IA who had all been prescribed a gabapentinoid at some point (to minimise confounding by indication).

**Methods:**

Our matched case–control study used linked national data from English primary care (Clinical Practice Research Datalink Aurum) and Hospital Episode Statistics. A cohort was constructed of adults with IA, contributing data 01/01/2004–31/03/2021, and ever prescribed oral gabapentinoids. Cases with an incident fracture post-cohort inclusion were ascertained and 1:5 risk set-matched (on age/gender/gabapentinoid type) with controls. Gabapentinoid prescription exposure was categorised as follows: (a) current (overlapping with fracture date); (b) recent (ending 1–60 days pre-fracture); and (c) remote (ending > 60 days pre-fracture). Conditional logistic regression models determined ORs with 95% CIs for fractures with current or recent vs. remote gabapentinoid use, adjusting for confounders.

**Results:**

A total of 2485 cases (mean age 63.0 years; 79.4% female) and 12,244 controls (mean age 62.7 years; 79.6% female) were included. Of cases: 1512 received gabapentin, 910 pregabalin, and 63 both drugs; 65.6% were remote, 5.5% recent, and 28.9% current users. In adjusted models, current gabapentinoid use had an increased risk of fracture (OR vs. remote: 1.36 [95% CI 1.22, 1.51]). Similar associations were seen with gabapentin (OR 1.38 [1.19, 1.60]) and pregabalin (OR 1.40 [1.18, 1.66]). Similar or higher levels of association were seen for all gabapentin/pregabalin doses except moderate/very high dose gabapentin. Associations were strongest in those starting gabapentinoids more recently.

**Conclusions:**

Our study suggests a modest association between current gabapentinoid use and fractures in patients with IA, after accounting for measured and time-invariant unmeasured confounding. Whilst other unmeasured confounding remains possible, given the absence of evidence for gabapentinoid efficacy in patients with IA who are particularly vulnerable to fractures, this highlights a need for efforts to deliver safer gabapentinoid prescribing in this population.

**Supplementary Information:**

The online version contains supplementary material available at 10.1186/s12916-024-03774-5.

## Background

Inflammatory arthritis (IA) is an umbrella-term grouping conditions causing persistent joint inflammation. Its three main forms—rheumatoid arthritis (RA), psoriatic arthritis (PsA), and axial spondyloarthritis (AxSpA)—affect > 1% of adults in England and North America [1–4], and are characterised by chronic pain [5], which has far-reaching impacts on patients’ lives [6]. Despite an absence of trials evaluating their efficacy for pain management in IA, the percentage of patients with IA prescribed gabapentinoids in England has risen substantially from < 1% in 2004 to approximately 10% in 2020 [7]. Substantial gabapentinoid use in IA is also seen in other European countries [8, 9]. This is despite the known harms associated with gabapentin and pregabalin (which include severe respiratory depression, withdrawal, misuse, and death) leading to both drugs being reclassified as class C controlled substances in the UK in 2018 (making it a criminal offence to possess them without a prescription) [10].

Of relevance to patients with IA are emerging observational study findings in non-IA populations that gabapentinoids associate with an increased risk of fractures (Additional file 1: Table S1 [11–22]), with osteoporosis and fractures both common IA comorbidities [23, 24]. The association between gabapentinoid use and fractures is not, however, consistently replicated across studies, with some (such as a case–control study examining vertebral, wrist, and hip fractures in 2196 cases and 8784 controls aged ≥ 50 years [20], and a cohort study examining non-traumatic fractures in 15,792 adults aged ≥ 50 years) [22] reporting an increased risk and others (such as a case–control study examining hip fractures in 4912 cases and 49,120 controls receiving haemodialysis) reporting no association [18]. Existing studies also show conflicting findings regarding whether any associations differ by gabapentinoid dose [15, 21] and type [13, 21]. A final complexity, inherent to all non-randomised studies of interventions, is the extent to which any observed associations could result from confounding.

In view of these issues, we conducted a case–control study to examine the relationship between gabapentinoids and fractures in patients with IA (a population particularly vulnerable to fractures, in whom this association has not previously been assessed), in which all people were prescribed a gabapentinoid at some point (minimising confounding by indication). The aims of our in-depth analysis in the Clinical Practice Research Datalink (CPRD) Aurum—a large electronic health record (EHR) database covering 20% of England—were to explore not only the overall association, but also whether it: (a) is present in both gabapentin and pregabalin users, (b) varies according to drug dose, and (c) is most marked (as with opioids) [25] in the initial period post-drug initiation.

## Methods

### Study design

This nested case–control study was conducted in CPRD Aurum, which contains routinely collected data from approximately 1500 GP practices and is representative of the English population by geographical spread, deprivation, age, and gender [26]. Aurum contains primary care data on diagnoses (coded using Read/SNOMED CT codes) and prescribed medicines. To optimise fracture ascertainment, we also used linked Hospital Episode Statistics Admitted Patient Care (HES APC) data, providing ICD-10 coded NHS hospital admission diagnoses and OPCS-4 coded procedures [27]. The May 2022 Aurum [28] and January 2022 HES APC [29] datasets were used. Data were extracted by JB from CPRD servers (accessed under Keele University’s multi-study licence).

### Study population

The study population comprised patients in English general practices contributing to CPRD Aurum: (a) fulfilling validated approaches for an RA, PsA, and AxSpA diagnosis [1]; (b) contributing data at any point from 01/01/2004 to 31/03/2021; (c) aged ≥ 18 years at first IA code; (d) ever receiving an oral gabapentinoid prescription; and (e) registered with their practice for at least 12 months prior to the date of their first IA Read/SNOMED code or first gabapentinoid prescription. This time-period (the start of 2004 to the latest date of available HES APC linked data in March 2021) was chosen as pregabalin was licenced in England in 2004 (gabapentin in 1993, although we felt it was appropriate to start the study for both types of gabapentinoid on the same date) and the time-period of nearly 17 years was sufficiently long to optimise the sample size. Cohort entry date was the latest of IA diagnosis date, gabapentinoid prescription date, and 01/01/2004. We used validated approaches to ascertain patients with RA, PsA, and AxSpA diagnoses. These have been described in detail previously [1]. Patients are classified as having RA if they have either: (1) ≥ one RA Read/SNOMED code and ≥ one synthetic disease-modifying anti-rheumatic drug (DMARD) prescription after the first RA code with no alternative DMARD indication (no Read/SNOMED code for an alternative indication for 5 years pre-first DMARD prescription) or (2) have ≥ two RA Read/SNOMED codes (on different dates, one of which must be deemed as being strong or fairly strong evidence of RA) and have no alternative diagnosis (alternative IA type) after the final code. Patients are classified as having AxSpA if they have two Read/SNOMED codes for AxSpA ≥ 7 days apart. Patients are classified as having PsA if they have a single PsA Read/SNOMED code. These classification approaches have high positive predictive values in primary care databases [30–32] and provide IA incidence/prevalence estimates in Aurum consistent with other datasets [1].

From this cohort, cases with a Read/SNOMED (Aurum) and/or ICD-10/OPCS-4 (HES APC) code for a diagnosis of/relevant procedure for an incident fracture were determined. Controls were then matched to cases using risk set sampling. For every case, up to five controls from individuals under observation in the study cohort on the date of the case fracture (index date) were randomly selected and matched on age at index date (within 5 years), gender, and gabapentinoid prescription type (gabapentin, pregabalin, or both at different times). This latter matching factor was included to ensure controls received the same type of gabapentinoid as their matched case. A control for a case on one date could become a control for another case occurring on a later index date, provided they remained in the study cohort. Controls were also at risk of later becoming a case. We considered the last IA Read/SNOMED code to represent their IA type. For the minority with codes for RA and PsA on the same date (as their last IA code), if a code for skin psoriasis was also present, we considered PsA to represent their IA type; if this was not present, we considered RA (most prevalent IA form) to represent their IA type. The date of the earliest IA code in their record was considered their diagnosis date.

### Outcome

We considered any bone fractures except those related to cancer or childbirth. They were ascertained using Read/SNOMED codes in Aurum and ICD-10 and OPCS-4 codes in HES APC. Code lists were developed by two rheumatologists (ICS and SH) working alongside a general practitioner (HT).

### Exposure and confounding variables

The exposure was the timing of the most recent gabapentinoid treatment period, categorised as follows: (a) current—prescription ended on/post-index date; (b) recent—prescription ended 1 to 60 days pre-index date; and (c) remote—prescription ended > 60 days pre-index date. The recent group was included as any gabapentinoid effect on fracture risk may persist for a short time after final dose, and some patients may (due to intermittent use) be using the gabapentinoid beyond the calculated prescription end date. This approach of prescription recency has been used previously in other studies of analgesic risks [33, 34]. Prescription duration was calculated based on the number of days for which the prescription was issued. Prescriptions within 56 days were combined into a treatment episode. Average daily gabapentinoid dose was calculated separately for gabapentin and pregabalin in current users for the most recent prescription treatment period; where the dose was missing this was inferred from available prescription information (Additional file 1: Table S2).

Confounding variables were identified by discussions between the primary care clinicians, rheumatologists, and epidemiologists within the research team based on their clinical experience and knowledge of the published literature. The following confounding variables were considered at index date: (a) age; (b) gender; (c) IA type; (d) IA duration; (e) previous fragility fracture(s) and/or presence of osteoporosis (coded diagnosis) and/or receipt of a bisphosphonate, raloxifene, or teriparatide at/pre-index date; (f) CKD stage III, IV, or V (coded diagnosis and/or eGFR < 60 on ≥ 2 occasions ≥ 3 months apart); (g) receipt of prescribed oral prednisolone at an average dose of ≥ 5 mg/day for ≥ 3 months in the 12 months pre-index date; and (h) receipt of a current opioid, benzodiazepine, anti-depressant, anti-epileptic drug (excluding gabapentinoids), or Z-drug (non-benzodiazepine hypnotic) prescription.

### Statistical analysis

Descriptive statistics summarised age, gender, IA types, the proportion with relevant comorbidities/prescriptions by case–control status, and the proportion with the presence/absence of the specified confounding variables by case–control and exposure status. Conditional logistic regression models compared gabapentinoid prescribing between cases and controls in multivariable models adjusting first, for age (modelled using a fractional polynomial), and second, for the specified confounding variables, with an odds ratio (OR) with 95% confidence intervals (CIs) determined for current and recent gabapentinoid use relative to remote use for cases compared to controls (unadjusted ORs were also calculated and reported in Supplementary Tables). Gender was not adjusted for, as it was perfectly balanced between cases and controls. Several secondary analyses were performed. First, analysis was undertaken in people exposed to gabapentin only and pregabalin only, to assess any difference in association between gabapentinoid types. Second, the exposure was considered by dose in current users, comprising low (< 900 mg/day), moderate (900 to 1799 mg/day), high (≥ 1800 to 2499 mg/day), and very high (≥ 2500 mg/day) for gabapentin (in keeping with previous studies) [35] and low (≤ 150 mg/day), moderate (151 to 300 mg/day), and high (> 300 mg/day) for pregabalin (in keeping with licenced dosing) [36]; individuals switching between gabapentin and pregabalin were excluded in this analysis. Third, the duration of current gabapentinoid use was stratified into deciles, with risk in each decile compared to risk in remote users (to evaluate whether the risk is greatest on recent initiation of the drug). Fourth, to understand potential effect modifiers, we fitted models with interactions between recency of gabapentinoid use and: (1) age (< 64 and ≥ 65 years); (2) presence of previous fragility fracture/osteoporosis diagnosis/osteoporosis medicine use; (3) current opioid use; and (4) long-term steroid use (defined as described previously). Fifth, the exposure was restricted to fragility fractures only (vertebrae, humerus, wrist, and hip fractures, alongside general fragility fracture codes). This is because gabapentinoids could cause fractures due to their central nervous system effects precipitating falls, and if this were the mechanism, they would be expected to particularly associate with fragility fractures. Finally, the analysis was repeated in patients with IA without a previous fracture. A 5% significance level was adopted throughout. Data management used R Studio (R version 4.1.3) and Stata (version 18). Statistical analyses used Stata (version 18).

### Guidelines

We adhered to the REporting of studies Conducted using Observational Routinely-collected Data (RECORD) extension of the STrengthening the Reporting of OBservational studies in Epidemiology (STROBE) guideline for the reporting of our study [37].

### Patient and public involvement

Public contributors with IA confirmed the need for this research as part of the funding application, reporting they felt uninformed about the risks of harms with pain medicines. They will also be involved in co-designing study dissemination messages that are easily understood by patients and the public.

### Code lists

All code lists used in this study are publicly available online [38].

## Results

### Case and control characteristics

From the overall cohort of 19,831 patients with IA, 2485 cases and 12,244 controls were ascertained and included in the analysis (Additional file 1: Fig. S1). Cases and controls were similar with respect to age (mean 63.0 vs. 62.7 years), gender (79.4% vs. 79.6% female), IA types (81.7% vs. 79.8% RA; 15.0% vs. 16.4% PsA; 3.3% vs. 3.8% AxSpA), disease duration (median 6.0 vs. 6.2 years), presence of CKD (35.4% vs. 35.5%), and receipt of a prescribed anti-depressant (83.7% vs. 82.6%), anti-epileptic drug (11.6% vs. 9.9%), and Z-drug (33.7% vs. 30.1%) (Table 1). Proportionally more cases had previous fragility fracture(s)/coded osteoporosis diagnoses/were prescribed osteoporosis medicines (56.8% vs. 35.3%), received long-term oral steroids (23.5% vs. 16.5%), and received an opioid (38.9% vs. 27.5%) and benzodiazepine (57.2% vs. 52.4%) at index date (Table 1). With regard to the exposure (recency of gabapentinoid prescription), there were more cases and controls with a remote prescription at the index date (65.6% and 74.6%) than currently receiving a prescription (28.9% and 21.8%) or with a recent prescription (5.5% and 3.6%) (Table 2). There were 1512 cases receiving gabapentin only and 910 pregabalin only, with 63 having received both drugs. Evaluating the presence or absence of the included confounding variables by remote, recent, and current gabapentinoid prescription exposure status (Additional file 1: Table S3) showed that there were proportionally more older people that were remote users and younger people that were current users, and proportionally more current gabapentinoid users that were also current users of opioids, long-term prednisolone, benzodiazepines, anti-depressants, Z-drugs, and anti-epileptic drugs (with the opposite seen for remote gabapentinoid users). This supports the inclusion of these covariates as confounding variables in multivariable models.

**Table 1:** Demographic and clinical characteristics of cases and controls

Characteristic	Cases (*n* = 2485)	Controls (*n* = 12,244)
Age in years, mean (95% CI)	63.0 (62.5, 63.6)	62.7 (62.5, 63.0)
Female gender, *n* (%)	1973 (79.4%)	9750 (79.6%)
Inflammatory arthritis type		
Rheumatoid arthritis, *n* (%)	2031 (81.7%)	9771 (79.8%)
Psoriatic arthritis, *n* (%)	373 (15.0%)	2003 (16.4%)
Axial spondyloarthritis, *n* (%)	81 (3.3%)	470 (3.8%)
Disease duration in years, median (IQR)	6.0 (2.6, 8.8)	6.2 (2.7, 9.1)
Past fragility fracture or coded osteoporosis diagnosis or osteoporosis medicine prescription, *n* (%)	1412 (56.8%)	4319 (35.3%)
Presence of chronic kidney disease stage III/IV/V, *n* (%)	880 (35.4%)	4342 (35.5%)
Long-term oral prednisolone in past year, *n* (%)	585 (23.5%)	2019 (16.5%)
Current receipt of opioid prescription, *n* (%)	966 (38.9%)	3364 (27.5%)
Current receipt of anti-depressant prescription, *n* (%)	2080 (83.7%)	10,117 (82.6%)
Current receipt of benzodiazepine prescription, *n* (%)	1422 (57.2%)	6420 (52.4%)
Current receipt of anti-epileptic drug prescription, *n* (%)	289 (11.6%)	1217 (9.9%)
Current receipt of Z-drug prescription, *n* (%)	838 (33.7%)	3679 (30.1%)

Long-term oral prednisolone: receipt of prednisolone prescription at a dose of ≥5 mg for ≥3 months in 12 months pre-index date. Osteoporosis medicine: receipt of bisphosphonate, raloxifene, or teriparatide prescription at/pre-index date. Anti-epileptic drug: gabapentinoids are excluded from this

**Table 2:** Association of fractures with current and recent gabapentinoid use compared to remote use

Use status	Any gabapentinoid				Gabapentin only				Pregabalin only			
	**Cases (*n* = 2485)**	**Controls (*n* = 12,244)**	**Age adjusted OR**	**Fully adjusted OR**	**Cases (*n* = 1512)**	**Controls (*n* = 7299)**	**Age adjusted OR**	**Fully adjusted OR**	**Cases (*n* = 910)**	**Controls (*n* = 4353)**	**Age adjusted OR**	**Fully adjusted OR**
Remote	1631 (65.6%)	9130 (74.6%)	1.00	1.00	1047 (69.2%)	5677 (77.8%)	1.00	1.00	544 (59.8%)	3041 (69.9%)	1.00	1.00
Recent	136 (5.5%)	444 (3.6%)	1.72 (1.40, 2.10)	1.70 (1.38, 2.09)	83 (5.5%)	263 (3.6%)	1.75 (1.34, 2.29)	1.79 (1.35, 2.36)	48 (5.3%)	148 (3.4%)	1.90 (1.31, 2.74)	1.77 (1.21, 2.59)
Current	718 (28.9%)	2670 (21.8%)	1.53 (1.38, 1.69)	1.36 (1.22, 1.51)	382 (25.3%)	1359 (18.6%)	1.54 (1.34, 1.77)	1.38 (1.19, 1.60)	318 (35.0%)	1164 (26.7%)	1.55 (1.31, 1.82)	1.40 (1.18, 1.66)

Current: receiving gabapentinoid prescription at index date; recent: receiving gabapentinoid prescription 1 to 60 days pre-index date; remote: receiving gabapentinoid prescription >60 days pre-index date; multivariable models adjusted for age, gender, inflammatory arthritis type (RA, PsA, AxSpA), inflammatory arthritis duration, previous fragility fracture/presence of osteoporosis diagnosis/osteoporosis medicine use, presence of chronic kidney disease, receipt of long-term oral steroids (prednisolone at a dose of ≥5 mg/day for ≥3 months during year pre-index date), and current receipt of opioid, anti-depressant, benzodiazepine, anti-epileptic drug (excluding gabapentinoids), and Z-drug prescription at index date. Individuals who had at some time been prescribed gabapentin and pregabalin were excluded from the analysis of specific drugs

### Association between recency of gabapentinoid use and fractures

Current gabapentinoid use associated with a modest increased risk of fracture in unadjusted, age adjusted, and fully adjusted models (Table 2; Additional file 1: Table S4), with a fully adjusted OR of 1.36 (1.22, 1.51). Examining associations by gabapentinoid type revealed similar strengths of association with fracture for current gabapentin and pregabalin use relative to remote use in unadjusted, age adjusted, and fully adjusted models, with ORs from fully adjusted models of 1.38 (1.19, 1.60) with gabapentin and 1.40 (1.18, 1.66) with pregabalin (Table 2; Additional file 1: Table S4). Recent gabapentinoid use was more strongly associated with an increased risk of fracture than current use for any gabapentinoid, pregabalin, and gabapentin (Table 2; Additional file 1: Table S4) with ORs from fully adjusted models of 1.70 (1.38, 2.09), 1.79 (1.35, 2.36), and 1.77 (1.21, 2.59), respectively. No significant interactions were observed between the recency of gabapentinoid use and: (1) age; (2) previous fragility fractures/osteoporosis diagnosis/osteoporosis medicine use; (3) current opioid use; and (4) long-term steroid use (Additional file 1: Tables S5 to S8).

### Association by current gabapentin and pregabalin dose

In unadjusted (Additional file 1: Table S9) and age adjusted (Table 3) models, current gabapentin use was associated with an increased risk of fracture for all dosage categories except very high dose gabapentin. In fully adjusted models, increased risks were only observed with low (OR 1.65 [1.33, 2.05]) and high (OR 1.37 [1.01, 1.85]) dose current users. For pregabalin (Additional file 1: Table S9; Table 3) a modest increased risk of fracture was seen for low (fully adjusted OR 1.36 [1.07, 1.72]), moderate (fully adjusted OR of 1.42 [1.09, 1.85]), and high (fully adjusted OR 1.45 [1.10, 1.93]) dosing, with the magnitude of association similar between dosing groups.

**Table 3:** Association of fractures with current gabapentin or pregabalin use according to prescription dose

Use status		Gabapentin only				Pregabalin only			
		**Cases (*n* = 1512)**	**Controls (*n* = 7299)**	**Age adjusted OR**	**Fully adjusted OR**	**Cases (*n* = 910)**	**Controls (*n* = 4353)**	**Age adjusted OR**	**Fully adjusted OR**
Remote		1047 (69.3%)	5677 (77.8%)	1.00	1.00	544 (59.8%)	3041 (69.9%)	1.00	1.00
Recent		83 (5.5%)	263 (3.6%)	1.75 (1.34, 2.29)	1.78 (1.35, 2.35)	48 (5.3%)	148 (3.4%)	1.90 (1.32, 2.74)	1.78 (1.22, 2.59)
Current	Low (gabapentin: < 900 mg/day; pregabalin: ≤ 150 mg/day)	151 (10.0%)	464 (6.4%)	1.81 (1.48, 2.23)	1.65 (1.33, 2.05)	128 (14.1%)	482 (11.1%)	1.54 (1.22, 1.93)	1.36 (1.07, 1.72)
	Moderate (gabapentin: 900 to 1799 mg/day; pregabalin: 151 to 300 mg/day)	116 (7.7%)	479 (6.6%)	1.30 (1.04, 1.63)	1.20 (0.96, 1.52)	103 (11.3%)	363 (8.3%)	1.52 (1.18, 1.96)	1.42 (1.09, 1.85)
	High (gabapentin: 1800 to 2499 mg/day; pregabalin > 300 mg/day)	72 (4.8%)	246 (3.4%)	1.59 (1.19, 2.12)	1.37 (1.01, 1.85)	87 (9.6%)	319 (7.3%)	1.62 (1.24, 2.12)	1.45 (1.10, 1.93)
	Very high (gabapentin ≥ 2500 mg/day)	43 (2.8%)	170 (2.3%)	1.41 (0.98, 2.02)	1.17 (0.80, 1.71)	–	–	–	–

Current: receiving gabapentinoid at index date. Recent: receiving gabapentinoid prescription 1 to 60 days pre-index date. Remote: receiving gabapentinoid >60 days pre-index date. Fully adjusted model includes the following covariates: age, gender, inflammatory arthritis type, inflammatory arthritis duration, previous fragility fracture/presence of osteoporosis diagnosis/osteoporosis medicine use, presence of chronic kidney disease, receipt of long-term prednisolone (prednisolone at a dose of ≥5 mg/day for ≥3 months during the year pre-index date), and receipt of an opioid, anti-depressant, benzodiazepine, anti-epileptic drug (excluding gabapentinoids), and Z-drug prescription at index date. Individuals who have at some time been prescribed gabapentin and pregabalin are excluded from this analysis

### Association by duration of current gabapentinoid use

Considering the duration of current gabapentinoid use in deciles revealed that most people received gabapentinoid prescriptions long-term (Table 4), with the shortest duration of use deciles comprising 14 to 128 days (decile 1) and 129 to 341 days (decile 2), and the longest being 3449 to 7647 days (decile 10). In unadjusted and age adjusted models, associations with fracture were only observed with shorter use duration deciles 1 to 5, alongside decile 8 in the age adjusted model (Table 4; Additional file 1: Table S10). In fully adjusted models, associations (of different strengths) were only seen for deciles 1 to 5 with ORs comprising 2.40 (1.87, 3.08), 1.82 (1.40, 2.36), 1.33 (1.01, 1.75), 1.51 (1.16, 1.98), and 1.33 (1.01, 1.76).

**Table 4:** Association of fractures with current gabapentinoid use by duration of current use

User status		Cases (*n* = 2485)	Controls (*n* = 12,244)	Age adjusted OR	Fully adjusted OR
Remote		1631 (65.6%)	9130 (74.6%)	1.00	1.00
Recent		136 (5.5%)	444 (3.6%)	1.73 (1.41, 2.12)	1.71 (1.39, 2.11)
Current decile	1	106 (4.3%)	234 (1.9%)	2.61 (2.05, 3.31)	2.40 (1.87, 3.08)
	2	90 (3.6%)	249 (2.0%)	2.03 (1.58, 2.61)	1.82 (1.40, 2.36)
	3	70 (2.8%)	273 (2.2%)	1.46 (1.12, 1.91)	1.33 (1.01, 1.75)
	4	77 (3.1%)	260 (2.1%)	1.68 (1.29, 2.18)	1.51 (1.16, 1.98)
	5	71 (2.96%)	266 (2.2%)	1.53 (1.16, 2.00)	1.33 (1.01, 1.76)
	6	63 (2.5%)	275 (2.3%)	1.29 (0.98, 1.71)	1.23 (0.92, 1.63)
	7	60 (2.4%)	281 (2.3%)	1.21 (0.91, 1.61)	1.04 (0.77, 1.39)
	8	64 (2.6%)	272 (2.2%)	1.33 (1.01, 1.76)	1.12 (0.84, 1.49)
	9	57 (2.3%)	282 (2.3%)	1.15 (0.86, 1.54)	0.99 (0.73, 1.34)
	10	60 (2.4%)	278 (2.3%)	1.23 (0.92, 1.63)	1.08 (0.80, 1.45)

Deciles comprise 1 = 14 to 128 days, 2 = 129 to 341 days, 3 = 342 to 624 days, 4 = 626 to 952 days, 5 = 953 to 1329 days, 6 = 1330 to 1716 days, 7 = 1718 to 2181 days, 8 = 2186 to 2695 days, 9 = 2699 to 3446 days, and 10 = 3449 to 7647 days. Current: receiving gabapentinoid at index date. Recent: receiving gabapentinoid prescription 1 to 60 days pre-index date. Remote: receiving gabapentinoid >60 days pre-index date. Fully adjusted model includes the following covariates: age, gender, inflammatory arthritis type, inflammatory arthritis duration, previous fragility fracture/presence of osteoporosis diagnosis/osteoporosis medicine use, presence of chronic kidney disease, receipt of long-term prednisolone (prednisolone at a dose of ≥5 mg/day for ≥3 months during the year pre-index date), and receipt of an opioid, anti-depressant, benzodiazepine, anti-epileptic drug (excluding gabapentinoids), and Z-drug prescription at index date

### Association between recency of gabapentinoid use and fragility fractures

Restricting the outcome to fragility fractures led to a substantial reduction in sample sizes (Table 5). Current use of any gabapentinoid associated with a modest increased risk of fragility fracture in unadjusted, age adjusted, and fully adjusted models (Table 5; Additional file 1: Table S11), with a fully adjusted OR of 1.33 (1.16, 1.53). Examining associations by gabapentinoid type revealed an increased risk with current gabapentin but not current pregabalin use. ORs from fully adjusted models comprised 1.48 (1.20, 1.82) for gabapentin and 1.07 (0.82, 1.38) for pregabalin. Recent use of any gabapentinoid was also associated with an increased risk of fragility fractures in unadjusted, age adjusted, and fully adjusted models, with a fully adjusted OR of 1.65 (1.26, 2.17). Examining associations by gabapentinoid type revealed stronger increased risks of fragility fractures with recent gabapentin use in all models (fully adjusted OR 1.79 [1.21, 2.64]) than for recent pregabalin use (fully adjusted OR 1.33 [0.82, 2.14]).

**Table 5:** Association of fragility fractures with current and recent gabapentinoid use compared to remote use

Use status	Any gabapentinoid				Gabapentin only				Pregabalin only			
	**Cases (*n* = 1426)**	**Controls (*n* = 7042)**	**Age adjusted OR**	**Fully adjusted OR**	**Cases (*n* = 739)**	**Controls (*n* = 3663)**	**Age adjusted OR**	**Fully adjusted OR**	**Cases (*n* = 372)**	**Controls (*n* = 1846)**	**Age adjusted OR**	**Fully adjusted OR**
Remote	944 (66.2%)	5274 (74.9%)	1.00	1.00	511 (69.2%)	2907 (79.4%)	1.00	1.00	234 (62.9%)	1243 (67.3%)	1.00	1.00
Recent	83 (5.8%)	279 (4.0%)	1.65 (1.28, 2.14)	1.65 (1.26, 2.17)	41 (5.5%)	127 (3.5%)	1.87 (1.29, 2.70)	1.82 (1.23, 2.70)	26 (7.0%)	97 (5.3%)	1.44 (0.92, 2.28)	1.33 (0.82, 2.14)
Current	399 (28.0%)	1489 (21.1%)	1.50 (1.32, 1.71)	1.33 (1.16, 1.53)	187 (25.3%)	629 (17.2%)	1.72 (1.42, 2.08)	1.48 (1.20, 1.82)	112 (30.1%)	506 (27.4%)	1.16 (0.91, 1.49)	1.07 (0.82, 1.38)

Current: receiving gabapentinoid at index date. Recent: receiving gabapentinoid prescription 1 to 60 days pre-index date. Remote: receiving gabapentinoid >60 days pre-index date. Fully adjusted model includes the following covariates: age, gender, inflammatory arthritis type, inflammatory arthritis duration, previous fragility fracture/presence of osteoporosis diagnosis/osteoporosis medicine use, receipt of long-term prednisolone (prednisolone at a dose of ≥5 mg/day for ≥3 months during the year pre-index date), and receipt of an opioid, anti-depressant, benzodiazepine, anti-epileptic drug (excluding gabapentinoids), and Z-drug prescription at index date. Individuals who had at some time been prescribed gabapentin and pregabalin were excluded from the analysis of specific drugs

### Association between recency of gabapentinoid use and fractures in people without previous fractures

Restricting the analysis to those without previous fractures (Additional file 1: Table S12) demonstrated minimal associations between current any gabapentinoid use (fully adjusted OR 1.19 [1.03, 1.38]), current gabapentin use (fully adjusted OR 1.20 [0.97, 1.48]), and current pregabalin use (fully adjusted OR 1.18 [0.93, 1.51]) and fracture. However, the sample size for these subgroups was substantially smaller than in the primary analysis.

## Discussion

Our nested case–control study has involved an in-depth analysis of the risk of fractures with gabapentinoids in patients with IA. It was conducted in a nationally representative, primary care database (which includes approximately 1500 general practices) and used linked hospital admission data to optimise fracture ascertainment. It used two approaches to reduce the risk of confounding that is inherent to non-randomised studies of interventions, namely comparing the risk of fractures in current to remote gabapentinoid users (minimising confounding by indication) and adjusting for confounding variables in regression models. It suggests a 36% increase in the odds of fractures in patients with IA currently receiving gabapentinoids (compared to those receiving them in the remote past), with an increase in the odds ranging from 22 to 51% also reasonably compatible with these data. Whilst it is not possible to determine causality in observational studies, when this finding is considered with the other known harms of gabapentinoids, their widespread prescribing in IA (received by an estimated 10% of patients with IA in Aurum in 2020 [7]), and the absence of any trial evidence for their efficacy in this population, we consider that our study highlights a need to reappraise the pharmacologically focused approach to IA pain care.

Our case–control study is the first to examine the risk of fractures with gabapentinoids in patients with IA. We identified 12 published observational studies examining the risk of fractures with gabapentinoids in our literature review (Additional file 1: Table S1); none of these examined risk in patients with IA. Six studies evaluated fracture risk with gabapentinoid use vs. non-use. One case–control study reported no statistically significant association with hip fractures in people receiving haemodialysis [18], but the remainder reported statistically significant associations with fractures in other populations. One cohort study reported an increased risk of a composite outcome of falls or fractures in US veterans with gabapentin [17]. One case–control study reported an increased risk of a composite outcome of “injuries” (including fractures) with pregabalin [39]. One case–control study reported an increased risk of vertebral, wrist, or hip fractures in older adults with gabapentin [20]. One cohort study reported an increased risk of fractures requiring an emergency room visit or hospitalisation in adults receiving haemodialysis with gabapentin at a dose of > 300 mg/day (considered “high-dose” in the context of end-stage renal disease) but not other dose categories, with no association seen with pregabalin [21]. One cohort study reported an increased risk of non-traumatic fractures with gabapentin (but also a range of other anti-epileptic drugs that were evaluated) [22]. Taken together with our study, these findings suggest that it is likely there is a relationship between gabapentinoid use and fractures.

There are two key potential mechanisms by which gabapentinoids could lead to fractures. The first mechanism is that their central nervous system (CNS) effects (with dizziness, ataxia, and abnormal co-ordination listed as common/very common side-effects of gabapentin and pregabalin) [40] could precipitate falls or other traumatic events that lead to fractures. Two studies identified in our literature review evaluated the relationship between gabapentinoid use and risks of fractures and falls separately, with contrasting findings: George et al. reported an increased risk of fractures (but not falls) with gabapentinoid use, compared to nortriptyline use, in 195,207 older adults [11], and Muanda et al. reported an increased risk of hospitalisation due to falls (but not fractures) with higher vs. lower dose gabapentin use in 74,084 older adults with CKD [15]. As falls will be infrequently coded in Aurum, we indirectly examined this possibility by evaluating whether fracture risk was greatest in the time nearest to starting gabapentinoids (when CNS side-effects would be expected to be highest). Whilst we only observed an increased risk in shorter use deciles, as these still spanned 3.5 years following starting a gabapentinoid, it would point against a mechanism of action involving acute CNS effects occurring on drug initiation. The second mechanism is that gabapentinoids could detrimentally affect bone health. Some evidence supports this, with Kanda et al. reporting a deterioration of cancellous bone microstructure after 12 weeks in rats receiving gabapentin, which they postulated could effect bone mineral density with long-term use [41], and a cohort study examining the relationship between antiepileptic drug use and rates of hip bone mineral density loss in 4222 older males (mean follow-up 4.6 years) reporting that gabapentin users (compared with non-users of anti-epileptic drugs) had a 1.4- to 1.8-fold higher adjusted rate of annual bone mineral density loss [42].

Whilst our case–control study design optimised statistical power to examine the association between gabapentinoid use and the relatively rare outcome of fractures, it meant we could not estimate incidence rates and absolute risks of fractures, which makes interpreting the clinical implications of our principal study finding (a 36% increase in the odds of fractures in patients with IA currently receiving gabapentinoids compared to those receiving them in the remote past) challenging. Regardless of this, our study suggests a modest association between current gabapentinoid use and fractures in patients with IA, with the caveat that this could be explained by non-causal factors, particularly unmeasured and time-varying confounding.

One uncertainty that cannot be addressed in our study is whether the increased risk of fractures we observed with gabapentinoids in patients with IA is greater than in non-IA populations. Whilst it could be speculated this may be the case, owing to the a priori increased fracture risk of patients with IA [23, 24], this remains unknown. However, as IA pain management sits apart from pain management in other populations (owing to the presence of synovitis, joint damage, and excess nociplastic pain [43]) alongside the fact that gabapentinoids are commonly prescribed in IA (with a 2017/2018 prescription prevalence in England of approximately 9–10% in IA [7] vs. 3% in the general population [44]), we consider that our study’s findings are of particular importance to improving pain care in patients with IA.

It is notable that we did not observe a variation in the magnitude of the association between fractures and current gabapentin or pregabalin use across dose categories. This could be accounted for by two explanations. First, is that people who are most sensitive to CNS side-effects (precipitating falls/trauma) could stop gabapentinoids on dosage up-titration due to non-fracture adverse events (and therefore never reach a high-dose). Second, is that clinicians may not prescribe high doses to groups of patients most at risk of fractures. Our finding that the association between fractures and the duration of current gabapentinoid use is confined to shorter use durations could be explained by people that tolerate gabapentinoids better (and who are therefore at a lower risk of CNS effects) continuing the medication for longer.

Our study has several strengths. First, it was conducted in a large, primary care, EHR database, which provides information on gabapentinoid use and risks in a generalisable population sample. Second, we used both primary care record coding and linked HES APC data coding to ascertain the presence/absence of fractures, which will have reduced the risk of under-ascertainment of the fracture outcome. Third, we used validated approaches to determine people with IA. Fourth, we used a specific study design approach to reduce the risk of confounding by indication, namely comparing fracture risk in current vs. remote users; in addition we adjusted for measured confounding variables. It also has several limitations. First, unmeasured confounding remains possible, particularly in terms of age (where we could not find an adequate number of matches if matching more closely), disease duration, disease activity (not routinely recorded in primary care EHR databases, although the evidence that disease activity is related to fractures appears inconsistent [45, 46]), or disease severity (with no marker for this consistently available in Aurum). Second, time-varying residual confounding is also possible. Third, we have assumed that the absence of a coded clinical event means it did not occur. Whilst we anticipate that fractures would be well-coded, the confounding variables osteoporosis and CKD are likely to be less so (although there is no reason to suppose they will be differentially recorded in cases and controls). Fourth, people may use gabapentinoids intermittently, leading to misclassification of current users as recent users (and potentially even remote users). We suspect that this may be the case, and would account for why an increased fracture risk was observed in both current and recent use groups. Fifth, the case–control design means we cannot estimate incidence rates/absolute risks of fractures. Sixth, data on secondary care prescriptions are unavailable in Aurum, however, in England it would be unusual for a secondary care specialist to prescribe a gabapentinoid to a patient, and if initiating one they would ordinarily request that this is provided by the patient’s GP. Finally, our study was not powered to consider interactions. However, given the relatively large sample size and lack of clinically meaningful differences in OR patterns across strata in our models, we consider our conclusion of a lack of effect modification in relation to the examined variables is reasonable.

## Conclusions

Our study suggests a modest association between current gabapentinoid use and fractures in patients with IA, given its underlying assumptions. Although there is a difference between statistical and clinical significance, and causality cannot be proven (with residual confounding a possibility), when considered alongside the increased risk of fractures seen in patients with IA compared to the general population, the lack of trial data to support their use in this setting, and the other potential drug harms, an argument can be made that our principal finding—a 36% higher odds of fractures in those currently receiving gabapentinoids compared to those receiving gabapentinoids in the remote past—is a clinically meaningful one. Our study results call into question the commonplace practice of prescribing gabapentinoids to manage chronic pain in people with IA. Further research is needed to understand why gabapentinoid prescribing is prevalent in people with IA, and what steps can be taken to ensure that people with IA receive evidence-based pain care (which could involve a randomised controlled trial to evaluate gabapentinoid efficacy).

## Supplementary Information

Additional file 1: Tables S1–S12 and Figure S1. Table S1 Previous studies examining the association between fractures and gabapentinoids. Table S2 Approach to calculating gabapentinoid treatment duration and dose. Table S3 Covariate status by exposure categories. Table S4 Unadjusted estimates for the association of fractures with current and recent gabapentinoid use compared to remote use. Table S5 Association of fractures with gabapentinoids stratified by age. Table S6 Association of fractures with gabapentinoids stratified by presence/absence of osteoporosis diagnosis or medicine use/fragility fractures. Table S7 Association of fractures with gabapentinoids stratified by receipt of current opioid prescription status. Table S8 Association of fractures with gabapentinoids stratified by receipt of long-term prednisolone prescription status. Table S9 Unadjusted estimates for the association of fractures with current gabapentin or pregabalin use according to prescription dose. Table S10 Unadjusted estimates for the association of fractures with current gabapentinoid use by duration of current use. Table S11 Unadjusted estimates for the association of fragility fractures with current and recent gabapentinoid use compared to remote use. Table S12 Association of fractures with current and recent gabapentinoid use compared to remote use in patients without previous fractures. Fig. S1 Study flow diagram.

## Data Availability

Data may be obtained from a third party and are not publicly available. The data were obtained from the Clinical Practice Research Datalink (CPRD). CPRD data governance does not allow us to distribute patient data to other parties. Researchers may apply for data access at http://www.CPRD.com/. This study is based in part on data from the Clinical Practice Research Datalink (CPRD) obtained under licence from the UK Medicines and Healthcare products Regulatory Agency. The data is provided by patients and collected by the NHS as part of their care and support. The interpretation and conclusions contained in this study are those of the authors alone. HES data copyright © 2023, re-used with the permission of The Health & Social Care Information Centre. All rights reserved. The OPCS Classification of Interventions and Procedures, codes, terms and text is Crown copyright (2016) published by Health and Social Care Information Centre, also known as NHS Digital and licensed under the Open Government Licence available at www.nationalarchives.gov.uk/doc/open-government-licence/open-government-licence.htm.

## Abbreviations

AxSpA: Axial spondyloarthritis
CKD: Chronic kidney disease
CI: Confidence interval
CNS: Central nervous system
CPRD: Clinical Practice Research Datalink
DMARD: Disease-modifying anti-rheumatic drug
EHR: Electronic health record
HES APC: Hospital Episode Statistics Admitted Patient Care
IA: Inflammatory arthritis
OR: Odds ratio
PsA: Psoriatic arthritis
RA: Rheumatoid arthritis
RECORD: REporting of studies Conducted using Observational Routinely-collected Data
STROBE: STrengthening the Reporting of OBservational studies in Epidemiology
