# Supplementary material for: Gabapentinoid use and the risk of fractures in patients with inflammatory arthritis: nested case–control study in the Clinical Practice Research Datalink Aurum
Source: BMC Med. 2024 Dec 12;22:575. doi: 10.1186/s12916-024-03774-5 (PMC11636031; doi:10.1186/s12916-024-03774-5)
Supplement: Supplementary file 1 — Additional file 1: Tables S1–S12 and Figure S1. Table S1 Previous studies examining the association between fractures and gabapentinoids. Table S2 Approach to calculating gabapentinoid treatment duration and dose. Table S3 Covariate status by exposure categories. Table S4 Unadjusted estimates for the association of fractures with current and recent gabapentinoid use compared to remote use. Table S5 Association of fractures with gabapentinoids stratified by age. Table S6 Association of fractures with gabapentinoids stratified by presence/absence of osteoporosis diagnosis or medicine use/fragility fractures. Table S7 Association of fractures with gabapentinoids stratified by receipt of current opioid prescription status. Table S8 Association of fractures with gabapentinoids stratified by receipt of long-term prednisolone prescription status. Table S9 Unadjusted estimates for the association of fractures with current gabapentin or pregabalin use according to prescription dose. Table S10 Unadjusted estimates for the association of fractures with current gabapentinoid use by duration of current use. Table S11 Unadjusted estimates for the association of fragility fractures with current and recent gabapentinoid use compared to remote use. Table S12 Association of fractures with current and recent gabapentinoid use compared to remote use in patients without previous fractures. Fig. S1 Study flow diagram. [file 12916_2024_3774_MOESM1_ESM.docx]

**Gabapentinoid Use and the Risk of Fractures in Patients with Inflammatory Arthritis: Nested Case-Control Study in the Clinical Practice Research Datalink Aurum**

**Supplementary Tables and Figure**

**Table S1. Previous Studies Examining the Association between Fractures and Gabapentinoids**

| **Study (Year)** | **Design** | **Region** | **Size** | **Population** | **Exposure(s)** | **Outcome(s)** | **Key Results** | **Methods to Address Confounding** |
| --- | --- | --- | --- | --- | --- | --- | --- | --- |
| George et al (2023) | Cohort. | North America | 195,207 | Older adults, with depression, anxiety, or peripheral neuropathy, dispensed ≥ one of study medicines between 2008 and 2018. | Nortriptyline, Amitriptyline, Venlafaxine, Duloxetine, Fluoxetine, Paroxetine, Sertraline, Citalopram, Escitalopram or Gabapentin. | ICD coded fractures. | In adjusted model gabapentin associated with increased risk of fractures compared to nortriptyline (HR 1.30; 95% CI 1.22, 1.39). | Multivariable models. |
| Schousboe et al (2023) | Cross-sectional. | North America. | 11,822 | People receiving DEXA with vertebral fracture assessment (VFA). | Liver enzyme inducing and non-inducing anticonvulsants (later including gabapentin) | Prevalent vertebral fracture on VFA | No association between use of non-LEI anticonvulsants for <2 years or ≥2 years (relative to no use) and fractures. | Multivariable models. |
| Jørgensen et al (2023) | Cohort. | Denmark | 48,272 | Adults receiving incident gabapentin or pregabalin prescription from 1996 to 2018 without previous fragility fractures. | gabapentin or pregabalin | ICD coded fragility fractures. | No difference in short-term (30, 90, and 180 days) risk of fragility fractures between patients initiating gabapentin or pregabalin. | Active comparator study; propensity scores. |
| Shah et al (2021) | Cohort. | North America | 47,964 | Chronic pain and anxiety disorders in 2016, with continuous enrolments from 2015 to 2016, prescribed any combination of opioid, benzodiazepine, gabapentinoid and SSRI/SNRI in 2017 for ≥7 days. | Any combination of seven drug regimens (benzodiazepine + opioid; benzodiazepine + gabapentinoid; benzodiazepine + SSRI/SNRI; opioid + gabapentinoid; opioid + SSRI/SNRI; gabapentinoid + SSRI/SNRI; ≥3 drug classes). | ICD-10 coded falls and fractures | Statistically significantly reduced risk of fractures with benzo + GABA relative to benzo/opioid (HR 0.76; 95% CI 0.59, 0.98) but not for GABA + SSRI/SNRI or opioid + GABA. This was not statistically significant in propensity score analysis. | Multivariable model; propensity scores. |
| Muanda et al (2022) | Cohort. | North America. | 74,084 | Older adults with CKD newly starting gabapentin or pregabalin. | Higher vs. lower dose gabapentin or pregabalin. | Composite outcome of encephalopathy, fall, fracture or hospital admission with respiratory depression within 30 days of starting new prescription for gabapentin or pregabalin. | Statistically significant increased risk of composite outcome with higher vs lower dose gabapentinoid use (risk ratio 1.27; 95% CI 1.13, 1.42) but not hospitalisation with fracture alone (risk ratio 1.14; 95% CI 0.91, 1.43). | Comparing higher to lower dose gabapentinoid use; propensity scores; e value analysis; use of negative control outcome. |
| Pisa et al (2021) | Cohort. | Finland | 5,522 | Adults with Alzeimer’s disease newly starting anti-epileptic drugs. | Anti-epileptic drugs, which include gabapentin, pooled. | ICD coded incident hip fracture. | Statistically significant increased risk of hip fracture in users vs. non-users (HR 1.17, 95% CI 1.05, 1.30). | Matching; propensity scores. |
| Rentsch et al (2020) | Cohort. | North America | 140,310 exposed; 431,408 unexposed. | Adults born between 1945 and 1965 with at least 1 outpatient visit on/after 1/10/1999. | Gabapentin. | ICD coded falls or fractures. | Statistically significant increased risks of fall/fracture (RR 1.35; 95% CI 1.28, 1.44); risk increased with higher dosing. | Propensity score matching. |
| Vangala et al (2020) | Case-control. | North America. | 4912 cases; 49,120 controls. | Adults dependent on haemodialysis. | Gabapentin and pregabalin. | ICD coded hip fractures. | No association between gabapentinoid use (relative to no use) and hip fractures. | Risk set matching; multivariable models. |
| Musich et al (2020) | Cohort | North America | 209,947 | Older adults with 1 year continuous medical and drug plan enrolment during 2017 and opioid use of ≥2 prescriptions for ≥15 days. | Concurrent use of CNS-active medications including gabapentinoids. | Coded diagnoses of injurious falls/fractures | Statistically significant increased risk of falls or fractures with use of >2 CNS medicines with opioids compared to opioids alone | Multivariable models. |
| Cheng et al (2019) | Case-control. | Taiwan | 2,196 cases; 8,784 controls. | Older adults. | Anti-epileptic drugs, which include gabapentin. | ICD coded vertebral, wrist, and hip fractures. | Statistically significant increased risk of fractures with current gabapentin use (OR 1.79; 95% CI 1.01, 3.18). | Matching; multivariable models. |
| Ishida et al (2018) | Cohort | North America | 140,899 | Adults receiving haemodialysis. | Gabapentin or pregabalin. | ICD coded fractures requiring emergency room visit or hospitalisation. | Increased risk of fractures with high dose gabapentin >300mg/day, but not other dose categories, relative to no use (HR 1.38; 95% CI 1.18, 1.61). No association between pregabalin and fractures. | Multivariable models. |
| Jetté et al (2011) | Cohort | Canada | 15,792 | Older adults. | Anti-epileptic drugs, which include gabapentin. | ICD coded non-traumatic fractures. | Statistically significantly increased risk of fractures with current gabapentin users relative to non-users (OR 1.49; 95% CI 1.10, 2.02), and most other anti-epileptic drugs. | Matching; multivariable models. |

Pubmed searched on the 14/2/24 with the search terms “fracture” AND “gabapentinoid” OR “gabapentin” OR “pregabalin”. From 99 citations, the above 12 studies considered the risk of fractures with gabapentinoids. No additional studies from their reference lists were identified.

**Table S2. Approach to Calculating Gabapentinoid Treatment Duration and Dose**

| **Duration** | **Dose** |
| --- | --- |
| Treatment duration was calculated by using the “numdays” variable provided in the CPRD data. If missing, we imputed with mean duration per patient per product if duration was between 7 and 56 days. If still missing, we imputed with mean duration per person (across all products) if duration was between 7 and 56 days. If still missing, we imputed with mean duration per patient per product. If still missing, we imputed with mean duration per product. Treatment episodes were created by joining prescriptions with a calculated end date less than 56 days before the next issue date. The duration of treatment used in analysis was the length of treatment episode in those who were current gabapentinoid users at the index date. The period of 56 days between prescriptions to indicate a new treatment episode was chosen to reflect two standard prescription periods of 28 days, as this was felt to represent a likely break in continuous treatment. | Where daily dose was missing, this was replaced with the total quantity prescribed, multiplied by strength of formulation, and divided by duration of treatment. Oral solutions were converted from mg/ml to estimated mg prescribed per day. We did not encounter missing quantity or strength values. The upper and lower limits of possible daily doses were constrained based on British National Formulary dosing (300mg and 4,800mg for Gabapentin; 50mg and 600mg for Pregabalin). |

**Table S3. Covariate Status by Exposure Categories**

| **Covariate** | | **Gabapentinoid Use Exposure Category** | | |
| --- | --- | --- | --- | --- |
|  |  | **Remote** | **Recent** | **Current** |
| *Age ≥65* | *Present* | 5,195 (74.7%) | 270 (3.9%) | 1,490 (21.4%) |
|  | *Absent* | 5,566 (71.6%) | 310 (4.0%) | 1,898 (24.4%) |
| *Osteoporosis diagnosis or medicine use/past fragility fracture* | *Present* | 4,126 (72.0%) | 224 (3.9%) | 1,381 (24.1%) |
|  | *Absent* | 6,635 (73.7%) | 356 (4.0%) | 2,007 (22.3%) |
| *Current opioid use* | *Present* | 2,559 (59.1%) | 183 (4.2%) | 1,588 (36.7%) |
|  | *Absent* | 8,202 (78.9%) | 397 (3.8%) | 1,800 (17.3%) |
| *Long-term prednisolone* | *Present* | 1,788 (68.7%) | 124 (4.8%) | 692 (26.6%) |
|  | *Absent* | 8,973 (74.0%) | 456 (3.8%) | 2,696 (22.2%) |
| *Benzodiazepine use* | *Present* | 5,610 (71.5%) | 310 (4.0%) | 1,922 (24.5%) |
|  | *Absent* | 5,151 (74.8%) | 270 (3.9%) | 1,466 (21.3%) |
| *Antidepressant use* | *Present* | 8,838 (72.5%) | 461 (3.8%) | 2,898 (23.8%) |
|  | *Absent* | 1,923 (76.0%) | 119 (4.7%) | 490 (19.4%) |
| *Z-drug use* | *Present* | 3,176 (70.3%) | 161 (3.6%) | 1,180 (26.1%) |
|  | *Absent* | 7,585 (74.3%) | 419 (4.1%) | 2,208 (21.6%) |
| *Anti-epileptic drug use* | *Present* | 1,068 (70.9%) | 61 (4.1%) | 377 (25.0%) |
|  | *Absent* | 9,693 (73.3%) | 519 (3.9%) | 3,011 (22.8%) |

**Table S4. Unadjusted Estimates for the Association of Fractures with Current and Recent Gabapentinoid Use Compared to Remote Use**

|  | **Any Gabapentinoid** | | | **Gabapentin Only** | | | **Pregabalin Only** | | |
| --- | --- | --- | --- | --- | --- | --- | --- | --- | --- |
| **Use Status** | **Cases**  **(n=2,485)** | **Controls**  **(n=12,244)** | **Unadjusted OR** | **Cases**  **(n=1,512)** | **Controls**  **(n=7,299)** | **Unadjusted OR** | **Cases**  **(n=910)** | **Controls**  **(n=4,353)** | **Unadjusted OR** |
| *Remote* | 1,631  (65.6%) | 9,130  (74.6%) | 1.00 | 1,047  (69.3%) | 5,677  (77.8%) | 1.00 | 544  (59.8%) | 3,041  (69.9%) | 1.00 |
| *Recent* | 136  (5.5%) | 444  (3.6%) | 1.72  (1.40, 2.10) | 83  (5.5%) | 263  (3.6%) | 1.76  (1.35, 2.30) | 48  (5.3%) | 148  (3.4%) | 1.89  (1.31, 2.73) |
| *Current* | 718  (28.9%) | 2,670  (21.8%) | 1.52  (1.38, 1.68) | 382  (25.3%) | 1,359  (18.6%) | 1.54  (1.34, 1.76) | 318  (35.0%) | 1,164  (26.7%) | 1.54  (1.31, 1.81) |

Current: receiving gabapentinoid prescription at index date; recent: receiving gabapentinoid prescription 1 to 60 days pre-index date; remote: receiving gabapentinoid prescription >60 days pre-index date. Individuals who had at some time been prescribed gabapentin and pregabalin were excluded from the analysis of specific drugs.

**Table S5. Association of Fractures with Gabapentinoids Stratified by Age**

|  | **Age <65 years** | | | | | **Age ≥65 years** | | | | |
| --- | --- | --- | --- | --- | --- | --- | --- | --- | --- | --- |
| **User Status** | **Cases**  **(n=1,284)** | **Controls**  **(n=6,490)** | **Unadjusted OR** | **Age Adjusted OR** | **Fully Adjusted OR** | **Cases**  **(n=1,201)** | **Controls**  **(n=5,754)** | **Unadjusted OR** | **Age Adjusted OR** | **Fully Adjusted OR** |
| *Remote* | 818  (63.7%) | 4,748  (73.2%) | 1.00 | 1.00 | 1.00 | 813  (67.7%) | 4,382  (76.2%) | 1.00 | 1.00 | 1.00 |
| *Recent* | 73  (5.7%) | 237  (3.7%) | 1.81  (1.38, 2.39) | 1.81  (1.38, 2.39) | 1.76  (1.33, 2.34) | 63  (5.3%) | 207  (3.6%) | 1.92  (1.38, 2.67) | 1.73  (1.23, 2.43) | 1.71  (1.20, 2.43) |
| *Current* | 393  (30.6%) | 1,505  (23.2%) | 1.54  (1.34, 1.76) | 1.54  (1.35, 1.76) | 1.33  (1.16, 1.53) | 325  (27.1%) | 1,165  (20.3%) | 1.80  (1.47, 2.22) | 1.63  (1.30, 2.03) | 1.47  (1.16, 1.86) |

**Table S6. Association of Fractures with Gabapentinoids Stratified by Presence/Absence of Osteoporosis Diagnosis or Medicine Use/Fragility Fractures**

|  | **Osteoporosis Diagnosis or Medicine Use/Past Fragility Fracture Present** | | | | | **Osteoporosis Diagnosis or Medicine Use/Past Fragility Fracture Absent** | | | | |
| --- | --- | --- | --- | --- | --- | --- | --- | --- | --- | --- |
| **User Status** | **Cases**  **(n=1,412)** | **Controls**  **(n=4,319)** | **Unadjusted OR** | **Age Adjusted OR** | **Fully Adjusted OR** | **Cases**  **(n=1,073)** | **Controls**  **(n=7,925)** | **Unadjusted OR** | **Age Adjusted OR** | **Fully Adjusted OR** |
| *Remote* | 910  (64.5%) | 3,216  (74.5%) | 1.00 | 1.00 | 1.00 | 721  (67.2%) | 5,914  (74.6%) | 1.00 | 1.00 | 1.00 |
| *Recent* | 84  (6.0%) | 140  (3.2%) | 2.10  (1.58, 2.79) | 2.11  (1.58, 2.80) | 2.05  (1.53, 2.74) | 52  (4.9%) | 304  (3.8%) | 1.40  (1.03, 1.90) | 1.39  (1.02, 1.89) | 1.39  (1.02, 1.89) |
| *Current* | 418  (29.6%) | 963  (22.3%) | 1.56  (1.36, 1.79) | 1.57  (1.36, 1.80) | 1.42  (1.23, 1.63) | 300  (28.0%) | 1,707  (21.5%) | 1.44  (1.24, 1.67) | 1.45  (1.25, 1.67) | 1.30  (1.12, 1.51) |

**Table S7. Association of Fractures with Gabapentinoids Stratified by Receipt of Current Opioid Prescription Status**

|  | **Current Opioid Use** | | | | | **No Current Opioid Use** | | | | |
| --- | --- | --- | --- | --- | --- | --- | --- | --- | --- | --- |
| **User Status** | **Cases**  **(n=966)** | **Controls**  **(n=3,364)** | **Unadjusted OR** | **Age Adjusted OR** | **Fully Adjusted OR** | **Cases**  **(n=1,519)** | **Controls**  **(n=8,880)** | **Unadjusted OR** | **Age Adjusted OR** | **Fully Adjusted OR** |
| *Remote* | 521  (53.9%) | 2,038  (60.6%) | 1.00 | 1.00 | 1.00 | 1,110  (73.1%) | 7,092  (79.9%) | 1.00 | 1.00 | 1.00 |
| *Recent* | 61  (6.3%) | 122  (3.6%) | 1.94  (1.40, 2.68) | 1.94  (1.40, 2.68) | 1.87  (1.34, 2.62) | 75  (4.9%) | 322  (3.6%) | 1.53  (1.18, 1.99) | 1.53  (1.18, 1.99) | 1.58  (1.21, 2.07) |
| *Current* | 384  (39.8%) | 1,204  (35.8%) | 1.26  (1.08, 1.46) | 1.26  (1.09, 1.47) | 1.24  (1.06, 1.44) | 334  (22.0%) | 1,466  (16.5%) | 1.47  (1.28, 1.68) | 1.47  (1.28, 1.69) | 1.48  (1.29, 1.70) |

**Table S8. Association of Fractures with Gabapentinoids Stratified by Receipt of Long-Term Prednisolone Prescription Status**

|  | **Long-Term Prednisolone Use** | | | | | **No Long-Term Prednisolone** | | | | |
| --- | --- | --- | --- | --- | --- | --- | --- | --- | --- | --- |
| **User Status** | **Cases**  **(n=585)** | **Controls**  **(n=2,019)** | **Unadjusted OR** | **Age Adjusted OR** | **Fully Adjusted OR** | **Cases**  **(n=1,900)** | **Controls**  **(n=10,225)** | **Unadjusted OR** | **Age Adjusted OR** | **Fully Adjusted OR** |
| *Remote* | 353  (60.3%) | 1,435  (71.1%) | 1.00 | 1.00 | 1.00 | 1,278  (67.3%) | 7,695  (75.3%) | 1.00 | 1.00 | 1.00 |
| *Recent* | 45  (7.7%) | 79  (3.9%) | 2.27  (1.53, 3.35) | 2.27  (1.53, 3.35) | 2.26  (1.51, 3.38) | 91  (4.8%) | 365  (3.6%) | 1.53  (1.21, 1.94) | 1.53  (1.20, 1.94) | 1.53  (1.20, 1.96) |
| *Current* | 187  (32.0%) | 505  (25.0%) | 1.50  (1.22, 1.84) | 1.51  (1.23, 1.86) | 1.45  (1.17, 1.78) | 531  (20.0%) | 2,165  (21.2%) | 1.50  (1.34, 1.68) | 1.50  (1.34, 1.68) | 1.33  (1.19, 1.50) |

**Table S9. Unadjusted Estimates for the Association of Fractures with Current Gabapentin or Pregabalin Use According to Prescription Dose**

| **User status** | | **Gabapentin only** | | | **Pregabalin only** | | |
| --- | --- | --- | --- | --- | --- | --- | --- |
|  |  | **Cases**  **(n=1,512)** | **Controls**  **(n=7,299)** | **Unadjusted OR** | **Cases**  **(n=910)** | **Controls**  **(n=4,353)** | **Unadjusted OR** |
| *Remote* | | 1,047  (69.3%) | 5,677  (77.8%) | 1.00 | 544  (59.8%) | 3,041  (69.9%) | 1.00 |
| *Recent* | | 83  (5.5%) | 263  (3.6%) | 1.76  (1.35, 2.30) | 48  (5.3%) | 148  (3.4%) | 1.89  (1.31, 2.73) |
| *Current* | *Low (gabapentin: <900mg/day; pregabalin: ≤150mg/day)* | 151  (10.0%) | 464  (6.4%) | 1.82  (1.48, 2.23) | 128  (14.0%) | 482  (11.1%) | 1.52  (1.22, 1.91) |
|  | *Moderate (gabapentin: 900 to 1799mg/day; pregabalin: 151 to 300mg/day)* | 116  (7.7%) | 479  (6.6%) | 1.30  (1.04, 1.62) | 103  (11.3%) | 363  (8.3%) | 1.51  (1.17, 1.94) |
|  | *High (gabapentin: 1,800mg to 2499mg/day; pregabalin >300mg/day)* | 72  (4.8%) | 246  (3.4%) | 1.59  (1.19, 2.11) | 87  (9.6%) | 319  (7.3%) | 1.61  (1.23, 2.1) |
|  | *Very high (gabapentin ≥ 2,500mg/day)* | 43  (2.8%) | 170  (2.3%) | 1.39  (0.97, 2.30) | - | - | - |

Current: receiving gabapentinoid at index date. Recent: receiving gabapentinoid prescription 1 to 60 days pre-index date. Remote: receiving gabapentinoid >60 days pre-index date. Individuals who have at some time been prescribed gabapentin and pregabalin are excluded from this analysis.

**Table S10. Unadjusted Estimates for the Association of Fractures with Current Gabapentinoid Use by**

**Duration of Current Use**

| **User Status** | | **Cases**  **(n=2,485)** | **Controls**  **(n=12,244)** | **Unadjusted OR** |
| --- | --- | --- | --- | --- |
| *Remote* | | 1,631  (65.6%) | 9,130  (74.6%) | 1.00 |
| *Recent* | | 136  (5.5%) | 444  (3.6%) | 1.73  (1.42, 2.12) |
| *Current Decile* | *1* | 106  (4.3%) | 234  (1.9%) | 2.60  (2.05, 3.30) |
|  | *2* | 90  (3.6%) | 249  (2.0%) | 2.03  (1.58, 2.61) |
|  | *3* | 70  (2.8%) | 273  (2.2%) | 1.47  (1.12, 2.92) |
|  | *4* | 77  (3.1%) | 260  (2.1%) | 1.68  (1.29, 2.18) |
|  | *5* | 71  (2.9%) | 266  (2.2%) | 1.51  (1.15, 1.98) |
|  | *6* | 63  (2.5%) | 275  (2.3%) | 1.29  (0.98, 1.71) |
|  | *7* | 60  (2.4%) | 281  (2.3%) | 1.21  (0.91, 1.61) |
|  | *8* | 64  (2.6%) | 272  (2.2%) | 1.32  (1.00, 1.75) |
|  | *9* | 57  (2.3%) | 282  (2.3%) | 1.14  (0.85, 1.52) |
|  | *10* | 60  (2.4%) | 278  (2.3%) | 1.23  (0.92, 1.63) |

Deciles comprise: 1 = 14 to 128 days; 2 = 129 to 341 days; 3 = 342 to 624 days; 4 = 626 to 952 days, 5 = 953 to 1,329 days, 6 = 1,330 to 1,716 days, 7 = 1,718 to 2,181 days, 8 = 2,186 to 2,695 days, 9 = 2,699 to 3,446 days, 10 = 3,449 to 7,647 days.

**Table S11. Unadjusted Estimates for the Association of Fragility Fractures with Current and Recent Gabapentinoid Use Compared to Remote Use**

|  | **Any Gabapentinoid** | | | **Gabapentin Only** | | | **Pregabalin Only** | | |
| --- | --- | --- | --- | --- | --- | --- | --- | --- | --- |
| **Use Status** | **Cases**  **(n=1,426)** | **Controls**  **(n=7,042)** | **Unadjusted OR** | **Cases**  **(n=739)** | **Controls**  **(n=3,663)** | **Unadjusted OR** | **Cases**  **(n=372)** | **Controls**  **(n=1,846)** | **Unadjusted OR** |
| Remote | 944  (66.2%) | 5,274  (74.9%) | 1.00 | 511  (69.2%) | 2,907  (79.4%) | 1.00 | 234  (62.9%) | 1,243  (67.3%) | 1.00 |
| Recent | 83  (5.8%) | 279  (4.0%) | 1.64  (1.27, 2.13) | 41  (5.5%) | 127  (3.5%) | 1.87  (1.30, 2.70) | 26  (7.0%) | 97  (5.3%) | 1.42  (0.90, 2.24) |
| Current | 399  (28.0%) | 1,489  (21.1%) | 1.51  (1.32, 1.72) | 187  (25.3%) | 629  (17.2%) | 1.72  (1.42, 2.07) | 112  (30.1%) | 506  (27.4%) | 1.18  (0.92, 1.51) |

Current: receiving gabapentinoid at index date. Recent: receiving gabapentinoid prescription 1 to 60 days pre-index date. Remote: receiving gabapentinoid >60 days pre-index date. Individuals who had at some time been prescribed gabapentin and pregabalin were excluded from the analysis of specific drugs.

**Table S12. Association of Fractures with Current and Recent Gabapentinoid Use Compared to Remote Use in Patients Without Previous Fractures**

|  | **Any Gabapentinoid** | | | | **Gabapentin Only** | | | | **Pregabalin Only** | | | |
| --- | --- | --- | --- | --- | --- | --- | --- | --- | --- | --- | --- | --- |
| **Use Status** | **Cases**  **(n=1,234)** | **Controls**  **(n=11,336)** | **Age Adjusted OR** | **Fully Adjusted OR** | **Cases**  **(n=762)** | **Controls**  **(n=6,758)** | **Age Adjusted OR** | **Fully Adjusted OR** | **Cases**  **(n=434)** | **Controls**  **(n=4,042)** | **Age Adjusted OR** | **Fully Adjusted OR** |
| *Remote* | 878  (71.2%) | 8,491  (74.9%) | 1.00 | 1.00 | 565  (74.2%) | 5,255  (77.8%) | 1.00 | 1.00 | 288  (66.4%) | 2,859  (70.7%) | 1.00 | 1.00 |
| *Recent* | 60  (4.9%) | 413  (3.6%) | 1.39  (1.03, 1.88) | 1.38  (1.02, 1.87) | 37  (4.9%) | 245  (3.6%) | 1.50  (1.00, 2.24) | 1.50  (1.00, 2.27) | 19  (4.4%) | 137  (3.4%) | 1.33  (0.77, 2.30) | 1.24  (0.71, 2.17) |
| *Current* | 296  (24.0%) | 2,432  (21.5%) | 1.21  (1.04, 1.40) | 1.19  (1.03, 1.38) | 160  (21.0%) | 1,258  (18.6%) | 1.23  (1.00, 1.51) | 1.20  (0.97, 1.48) | 127  (29.3%) | 1,046  (25.9%) | 1.18  (0.93, 1.51) | 1.18  (0.93, 1.51) |

Current: receiving gabapentinoid prescription at index date; recent: receiving gabapentinoid prescription 1 to 60 days pre-index date; remote: receiving gabapentinoid prescription >60 days pre-index date; multivariable models adjusted for age, gender, inflammatory arthritis type (RA, PsA, AxSpA), inflammatory arthritis duration, previous fragility fracture/presence of osteoporosis diagnosis/osteoporosis medicine use, presence of chronic kidney disease, receipt of long-term oral steroids (prednisolone at a dose of ≥5mg/day for ≥3 months during year pre-index date), and receipt of an opioid, anti-depressant, benzodiazepine, anti-epileptic drug (excluding gabapentinoids), and Z-drug prescription at index date. Individuals who had at some time been prescribed gabapentin and pregabalin were excluded from the analysis of specific drugs.

**Figure S1. Study Flow Diagram**

Patients with acceptable data in Aurum practices in England that: (a) received a Read/SNOMED code for RA, PsA, and AxSpA; (b) contributed data at any point from 01/01/2004 to 31/03/2021; (c) were aged ≥18 years at first IA code; (d) ever receiving an oral gabapentinoid prescription; (e) registered with their practice for at least 12 months prior to the date of their first IA Read/SNOMED code or first gabapentinoid prescription.

N = 24,253

Excluded (does not meet algorithm):

RA: N = 3,418

PsA: N = 0

AxSpA: N = 730

Meet PsA algorithm.

N = 4,180

Meet AxSpA algorithm.

N = 1,165

Meet RA algorithm.

N = 14,486

Total patients satisfying algorithms, where meeting more than 1 algorithm allowed.

N = 19,831

Aurum data only used to define fractures in n=923 people with potential HES data, but with >20 potential HES ID matches or a match rank>1

Read/SNOMED/ICD-10/OPCS-4 code for diagnosis of fragility fracture following first IA and gabapentinoid code.

N = 1,426

Read/SNOMED/ICD-10/OPCS-4 code for diagnosis of incident fracture following first IA and gabapentinoid code.

N = 2,485

Matching

Cases = 1,426; Controls = 7,042.

Matching

Cases = 2,485; Controls = 12,244.

**Secondary analysis with fragility fractures only.**

**Primary analysis with any fracture.**

**Allocating IA type:** as patients were allowed to satisfy more than one algorithm, IA type was allocated by taking the latest IA code. For those that had an RA and PsA code on the same date being the latest IA code, PsA was taken as the IA type if there was a record of psoriasis at any time. If no record of psoriasis, then RA was taken to be the IA type. There were no other combinations of IA type when allocating the latest IA code as IA type.

**Acceptable Aurum data:** ‘Acceptable’ patients refer to patients coded as either Male or Female under Gender and coded 1 under Acceptable flag.

**Algorithms**

*Rheumatoid arthritis (RA) Algorithm:*

The algorithm classifies patients as having RA if they meet one of two criteria:

(1) have ≥ one RA Read code and ≥ one disease-modifying anti-rheumatic drug (DMARD) prescription after the first RA code with no alternative DMARD indication (no Read/SNOMED code for an alternative indication for 5 years pre-first DMARD prescription).

(2) have:

(a) ≥two RA Read/SNOMED codes (on different dates);

(b) no alternative diagnosis (alternative inflammatory arthritis type) after the final code; and

(c) a code from strength of evidence groups 1/2, as opposed to 3/4

*Psoriatic arthritis (PsA) algorithm:*

Single PsA Read/SNOMED code are evidence for a diagnosis.

*Axial spondylarthritis (AxSpA) algorithm:*

Two Read/SNOMED codes for AxSpA ≥ seven days apart.

**Acceptable linkage to HES APC:** ‘Acceptable’ patients refers to patients who have ≤ 20 HES Id’s and Match rank = 1.

**Matching criteria:**  Matching on a 5:1 basis, controls are matched to cases using risk set sampling. Matched on age at index (within 5 years age bands), gender and gabapentinoid type. A control for a case on one date could become a control for another case occurring on a later index date, provided they remained in the study cohort and were also at risk of becoming a case. Of those included in the final eligible cohort, some were included in the case control study multiple times as cases and/or controls whilst others were not included after matching.
